# Determinants of birth registration in sub-Saharan Africa: evidence from demographic and health surveys

**DOI:** 10.3389/fpubh.2023.1193816

**Published:** 2023-07-20

**Authors:** Richard Gyan Aboagye, Joshua Okyere, Abdul-Aziz Seidu, Bright Opoku Ahinkorah, Eugene Budu, Sanni Yaya

**Affiliations:** ^1^Department of Family and Community Health, Fred N. Binka School of Public Health, University of Health and Allied Sciences, Hohoe, Ghana; ^2^Department of Population and Health, University of Cape Coast, Cape Coast, Ghana; ^3^Department of Nursing, College of Health Sciences, Kwame Nkrumah University of Science and Technology, Kumasi, Ghana; ^4^REMS Consult Limited, Takoradi, Ghana; ^5^College of Public Health, Medical and Veterinary Sciences, James Cook University, Townsville, QLD, Australia; ^6^School of Public Health, Faculty of Health, University of Technology Sydney, Sydney, NSW, Australia; ^7^Research Unit, Korle Bu Teaching Hospital, Accra, Ghana; ^8^Faculty of Medicine, University of Parakou, Parakou, Benin

**Keywords:** birth, registration, demography, children, demographic and health survey

## Abstract

**Background:**

Birth registration is a crucial aspect of ensuring that children have access to their rights and benefits, including health care, education, and citizenship. In sub-Saharan Africa (SSA), birth registration rates remain low, with millions of children going unregistered each year. Understanding the predictors of birth registration among children in this sub-region is important for developing targeted interventions to improve registration rates. The study examines the predictors of birth registration among children in SSA.

**Methods:**

We performed a cross-sectional analysis of secondary data pooled from the Demographic and Health Survey of 17 countries conducted from 2015 to 2021. A weighted sample of 162,500 children was included in the final analysis. We summarized the proportion of birth registration among children in SSA using a forest plot. We utilized a multilevel binary logistic regression analysis to examine the predictors of birth registration. The results were presented using adjusted odds ratios (aOR) with 95% confidence intervals (CIs).

**Results:**

We found that 48.32% [48.15–48.49] of births in SSA were registered. The lowest and highest prevalence of birth registration were found in Ethiopia (2.70 [2.38–3.02]) and Sierra Leone (92.93 [92.36–93.50]), respectively. Increasing child’s age was found to be significantly associated with a higher likelihood of birth registrations, with those aged 4 years [aOR = 1.55; CI = 1.49, 1.62] having the highest odds of birth registration compared to those aged below 1 year. Children born to mothers with primary [aOR = 1.17; CI = 1.11, 1.24], secondary [aOR = 1.44; CI = 1.34, 1.54], and higher education [aOR = 1.71; CI = 1.48, 1.99] were more likely to be registered than those born to mothers who had no formal education. Also, children born in health facilities were more likely to be registered [aOR = 1.60; CI = 1.48, 1.74] than those born at home. The odds of birth registration were significantly higher among children whose mothers received assistance during delivery [aOR = 1.88; CI = 1.72, 2.04], those in the richest wealth index [aOR = 3.91; CI = 3.54, 4.33], and those in rural areas [aOR = 1.92; CI = 1.76, 2.10].

**Conclusion:**

There is low childbirth registration coverage in SSA. The predictors of this phenomenon are the child’s age, maternal level of education, wealth index, place of residence, sub-region, maternal age, place of delivery, assistance during delivery, marital status, and sex of household head. Interventions and policies developed to improve childbirth registration coverage in SSA should prioritize mothers with no formal education, those who deliver at home, those with low socioeconomic status, those living in female headed household, and adolescent mothers.

## Background

As social beings, the identity of every individual is critical to their existence and recognition. Birth registration has long been one of the key conduits through which an individual’s identity is made known. In this context, birth registration refers to the process of recording a child’s birth (1). This process enables individuals to have access to their rights and benefits, including health care, education, and citizenship (2, 3). Moreover, the United Nations regards birth registration as a fundamental human right of every child (4). Despite the relevance and positionality of birth registration as a human right, there are significant lapses in its coverage.

Worldwide, it is estimated that nearly 166 million children under age five (i.e., representing one in four children) are not formally registered, with another 237 million children under-five having no birth certificates (5). In sub-Saharan Africa (SSA), only 46% of children under-five are registered (6). This high level of unregistered children in SSA is perplexing as it has serious consequences for children and their families. For instance, the lack of birth registration can lead to denial of access to services and opportunities, and makes it difficult to track child mortality rates and ensure the protection of children’s rights (7). To accelerate efforts to improve birth registration, the African Union has set a goal to achieve universal birth registration by 2025 (8). This goal can only be achieved when there is a clear understanding of the factors that predict birth registration among children in SSA.

Indeed, there have been some studies conducted in individual sub-Saharan African countries that have identified a combination of demographic (i.e., mother’s age, education level, and wealth), geographic (i.e., location of the household and access to services), and socio-cultural (i.e., cultural beliefs and attitudes toward birth registration) factors that predict birth registrations (9–11). However, these previous studies do not demonstrate the regional dynamics in relation to birth registration and its predictors. Thus, suggesting a substantial knowledge gap that ought to be filled. Our study narrows this knowledge gap by examining the predictors of birth registration among children in SSA. The findings from this study are important for developing targeted interventions to improve registration rates.

## Materials and methods

### Data source and study design

This study involved a cross-sectional analysis of secondary data pooled from the Demographic and Health Survey (DHS) of 17 sub-Saharan African countries conducted from 2015 to 2021. The dataset used can be accessed via https://dhsprogram.com/data/available-datasets.cfm. The data were extracted from the kids recode (KR file) and the household member’s recode (PR file). According to Croft et al. (12), DHS is a comparable nationally representative survey undertaken regularly in over 90 countries to advance global understanding of health and population trends in developing countries. Data from the respondents were gathered by DHS using a descriptive cross-sectional approach. From the DHS, the respondents: men, women, and children who responded to structured questionnaires provided information on a range of socioeconomic and health indicators, including birth registration and certification (12, 13). DHS utilized a two-stage cluster sampling method, with the detailed sampling technique highlighted in the literature (14). A weighted sample of 162,500 children with completed observations on all variables of interest was included in the final analysis. In reporting this study, we relied on the Strengthening the Reporting of Observational Studies in Epidemiology (STROBE) guidelines (15).

### Variables

Birth registration was the outcome variable in the study. It was derived from the question that asked if children aged 0–4 years on the household roster have a birth certificate. The respondents were asked the question: Does (NAME) have a birth certificate? The response options were “has certificate,” “registered,” “neither,” and “do not know.” The “do not know” response option was dropped. Next, we recoded those whose response option was ‘neither’ as “no” and was assigned a value “0.” The remaining two response options: has certificate and registered were added together to generate a new response option “yes = 1”. We utilized the recoded response options: no and yes in the final analysis (3).

Eleven (11) explanatory variables were considered for inclusion into the study based on their availability in the DHS dataset as well their association with birth registration from previous studies (9, 16–18). We further segregated the variables into the individual level and the household/community level. The individual level variables consisted of child’s age, sex of child, mother’s age, educational level, marital status, place of delivery, and assistance during delivery. Household wealth index, sex of household head, place of residence, and geographical subregion were the household/community level variables. Table 1 contains the categories of the variables included in the study.

**Table 1:** Distribution of birth registration across the explanatory variables.

Variable	Weighted	Birth registration status		
	*N* (%)	No [95% CI]	Yes [95% CI]	*p* value
Child’s age (Years)				<0.001
<1	36,382 (22.4)	48.3 [47.2–49.4]	51.7 [50.6–52.8]	
1	34,043 (20.9)	43.9 [42.9–45.0]	56.1 [55.0–57.1]	
2	31,765 (19.6)	42.3 [41.3–43.4]	57.7 [56.6–58.7]	
3	31,654 (19.5)	41.2 [40.1–42.3]	58.8 [57.7–59.9]	
4	28,656 (17.6)	40.5 [39.4–41.6]	59.5 [58.4–60.6]	
Sex of child				0.327
Male	81,905 (50.4)	43.3 [42.3–44.3]	56.7 [55.7–57.7]	
Female	80,595 (49.6)	43.6 [42.7–44.6]	56.4 [55.4–57.3]	
Women’s age (years)				<0.001
15–19	9,881 (6.1)	52.1 [50.5–53.7]	47.9 [46.3–49.5]	
20–24	35,920 (22.1)	46.3 [45.2–47.3]	53.7 [52.6–54.8]	
25–29	43,288 (26.7)	43.1 [41.9–44.2]	56.9 [55.8–58.1]	
30–34	4,331 (21.1)	40.9 [39.8–42.1]	59.1 [57.9–60.2]	
35–39	24,560 (15.1)	40.1 [38.9–41.4]	59.9 [58.6–61.1]	
40–44	11,206 (6.9)	43.6 [42.1–45.2]	56.4 [54.8–57.9]	
45–49	3,314 (2.0)	42.2 [39.8–44.8]	57.8 [55.2–60.2]	
Level of education				<0.001
No education	58,599 (36.1)	44.3 [42.9–45.7]	55.7 [54.3–57.1]	
Primary	64,781 (39.9)	48.4 [47.4–49.5]	51.6 [50.5–52.6]	
Secondary	34,707 (21.3)	35.2 [34.0–36.4]	64.8 [63.6–66.0]	
Higher	4,413 (2.7)	24.6 [22.2–27.2]	75.4 [72.8–77.8]	
Marital status				<0.001
Never in union	8,362 (5.2)	48.7 [46.9–50.5]	51.3 [49.5–53.1]	
Married	115,719 (71.2)	40.2 [39.1–41.2]	59.8 [58.8–60.9]	
Cohabiting	26,987 (16.6)	52.5 [51.0–54.0]	47.5 [46.0–49.0]	
Widowed	2,023 (1.2)	46.3 [43.2–49.5]	53.7 [50.5–56.8]	
Divorced	3,336 (2.1)	55.5 [53.0–58.0]	44.5 [42.0–47.0]	
Separated	6,073 (3.7)	51.4 [49.5–53.3]	48.6 [46.7–50.5]	
Place of delivery				<0.001
Home	47,259 (29.1)	64.5 [63.1–65.9]	35.5 [34.1–36.9]	
Health facility	113,164 (69.6)	34.5 [33.7–35.4]	65.5 [64.6–66.3]	
Other	2,077 (1.3)	49.8 [46.9–52.8]	50.2 [47.2–53.1]	
Assistance during delivery				<0.001
No	52,517 (32.3)	64.7 [63.4–66.0]	35.3 [34.0–36.6]	
Yes	109,983 (67.7)	33.3 [32.5–34.1]	66.7 [65.9–67.5]	
Wealth index				<0.001
Poorest	38,422 (23.6)	56.9 [55.4–58.3]	43.1 [41.7–44.6]	
Poorer	35,352 (21.8)	49.5 [48.2–50.9]	50.5 [49.1–51.8]	
Middle	32,617 (20.1)	42.4 [41.1–37.0]	57.6 [56.2–58.9]	
Richer	30,101 (18.5)	35.6 [34.2–37.0]	64.4 [63.0–65.8]	
Richest	26,008 (16.0)	25.8 [24.4–27.2]	74.2 [72.8–75.6]	
Sex of household head				<0.001
Male	128,737 (79.2)	42.6 [41.6–43.6]	57.4 [56.4–58.4]	
Female	33,763 (20.8)	46.7 [45.6–47.9]	53.3 [52.1–54.4]	
Place of residence				<0.001
Urban	46,733 (28.8)	35.8 [34.4–37.3]	64.2 [62.7–65.6]	
Rural	115,767 (71.2)	46.5 [45.4–47.7]	53.5 [52.3–54.6]	
Subregion				<0.001
Central Africa	40,485 (24.9)	38.7 [36.9–40.4]	61.3 [59.6–63.1]	
Eastern Africa	41,361 (25.5)	64.4 [62.6–66.1]	35.6 [33.9–37.4]	
Southern Africa	28,961 (17.8)	51.5 [49.8–53.2]	48.5 [46.8–50.2]	
Western Africa	51,693 (31.8)	26.0 [24.6–27.3]	74.0 [72.7–75.4]	

### Statistical analyses

After data cleaning in each country’s dataset, we appended the dataset from all 17 countries in SSA included in the study. We summarized the proportion of birth registration among the children in SSA using a forest plot. We examined the distribution of birth registration across the explanatory variables using cross-tabulations. Next, the Pearson chi-square test of independence was used to determine the association between birth registration and the explanatory variables at *p* < 0.05. We utilized a multilevel binary logistic regression analysis to examine the predictors of birth registration. The first model (Model O) revealed the variance in birth registration attributed to the primary sampling unit (PSU) by being an empty model with no explanatory variables. Model I only included individual-level variables, while Model II included household/community level variables. Model III contained all the explanatory variables. The results were presented using adjusted odds ratios (aOR) with 95% confidence intervals (CIs). The “melogit” program in Stata was used to execute the multilevel regression models. To account for disproportionate sampling and non-response, the “svyset” command was used, and weighting was done to account for the intricate nature of DHS data.

### Ethical consideration

We did not seek ethical clearance since the dataset used is already available in the public domain. We complied with all the ethical guidelines regarding the use of a secondary dataset for publication after permission to use the dataset was granted by the Monitoring and Evaluation to Assess and Use Results Demographic and Health Surveys (MEASURE DHS).

### Results

#### Prevalence of birth registration in sub-Saharan Africa

**Figure 1:**
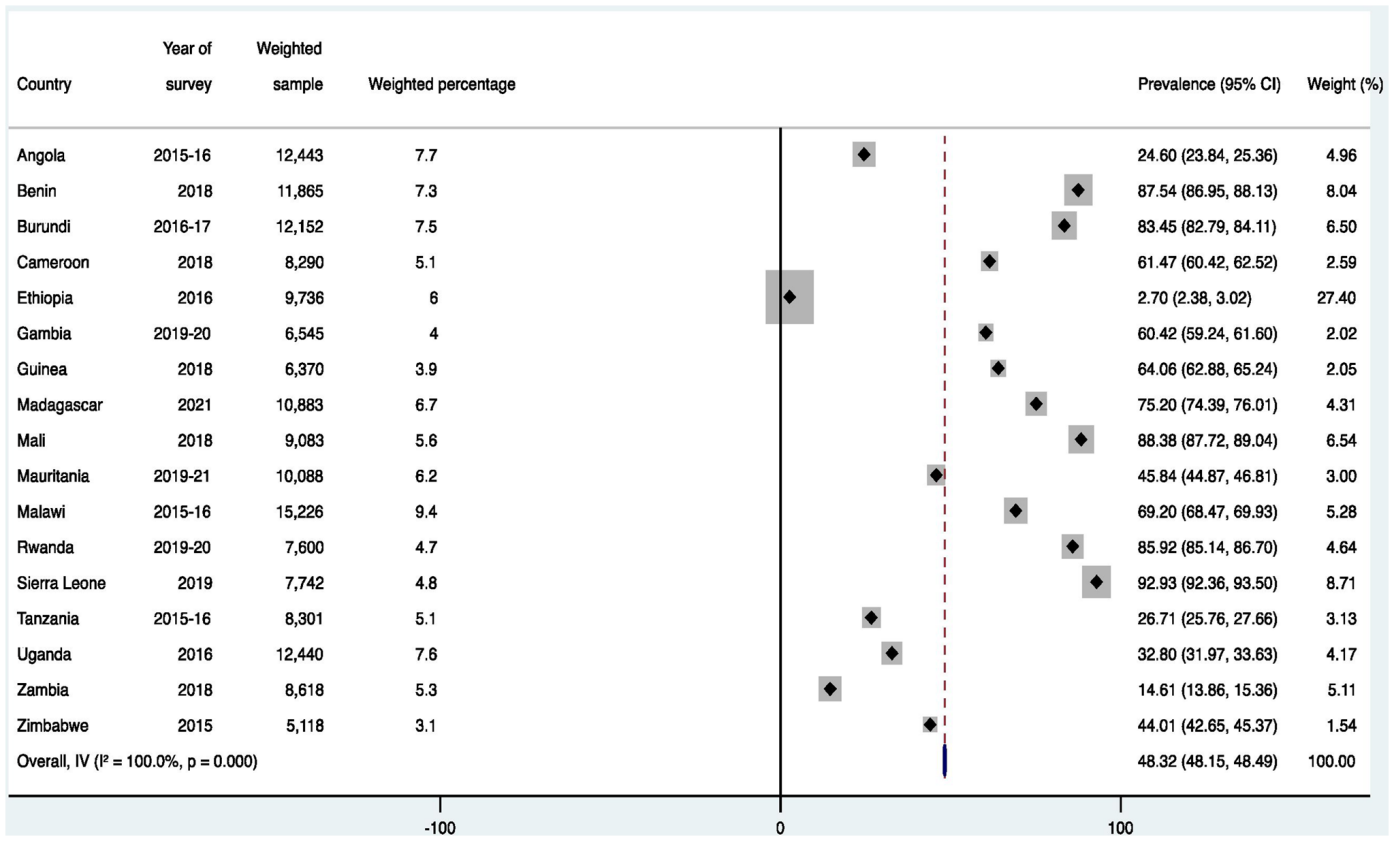
Prevalence of birth registration among children under five in sub-Saharan Africa.

Figure 1 presents results on the prevalence of birth registration in SSA. The results show that 48.32% [48.15–48.49] of births in SSA were registered. The lowest and highest prevalence of birth registration were in Ethiopia (2.70% [2.38–3.02]) and Sierra Leone (92.93% [92.36–93.50]), respectively.

#### Distribution of birth registration across the explanatory variables

Table 1 presents the distribution of birth registration across the various explanatory variables. The proportion of birth registration was highest among children aged 4 years (59.5%) and male sex children (56.7%). Birth registration was prevalent among children whose mothers were aged 35–39 years (59.9%), those whose mothers had attained a higher educational level (75.4%), and those whose mothers were married (59.8%).

Regarding the place of delivery, women who delivered at a health facility (65.5%) had a higher proportion of registered births compared to those who delivered at home (35.5%). Similarly, birth registration was higher among children born to mothers who received assistance during delivery (66.7%). The findings also show that there is a high proportion of birth registration among children from households with richest wealth index (74.2%). A greater proportion of birth registration was recorded among those in urban areas (64.2%) and male-headed households (57.4%). There were significant differences in the proportion of birth registration in the sub-region with the Western sub-region having the highest (74%) while births in the Eastern part recorded the least births registered (35.6%). All the explanatory variables except for the sex of the child were significantly associated with birth registration at *p* < 0.05.

#### Predictors of birth registration among children in sub-Saharan Africa

##### Fixed effect results

In Table 2, we present the results from the regression analysis of the predictors of birth registration among children in SSA. Except for the sex of the child, all the other explanatory variables significantly predicted birth registration. Increasing child age was found to be significantly associated with a higher likelihood of birth registration, with those aged 4 years [aOR = 1.55; 95%CI = 1.49, 1.62] having the highest odds of birth registration compared to those aged below 1 year.

**Table 2:** Predictors of birth registration among children in sub-Saharan Africa.

Variables	Model O	Model I aOR [95% CI]	Model II aOR [95% CI]	Model III aOR [95% CI]
Fixed effect				
Child’s age(years)				
<1		1.00		1.00
1		1.22^***^ [1.18, 1.28]		1.26^***^ [1.21, 1.31]
2		1.34^***^ [1.29, 1.40]		1.39^***^ [1.34, 1.45]
3		1.43^***^ [1.37, 1.48]		1.50^***^ [1.44, 1.57]
4		1.48^***^ [1.42, 1.54]		1.55^***^ [1.49, 1.62]
Sex of child				
Male		1.00		1.00
Female		1.00 [0.98, 1.03]		1.01 [0.98, 1.04]
Women’s age (years)				
15–19		1.00		1.00
20–24		1.11^**^ [1.04, 1.19]		1.11^**^ [1.04, 1.19]
25–29		1.21^***^ [1.13, 1.30]		1.11^**^ [1.03, 1.19]
30–34		1.31^***^ [1.22, 1.40]		1.22^***^ [1.13, 1.32]
35–39		1.37^***^ [1.28, 1.47]		1.28^***^ [1.19, 1.38]
40–44		1.25^***^ [1.15, 1.36]		1.19^***^ [1.09, 1.31]
45–49		1.34^***^ [1.19, 1.50]		1.28^***^ [1.13, 1.44]
Level of education				
No education		1.00		1.00
Primary		0.74^***^ [0.70, 0.77]		1.17^***^ [1.11, 1.24]
Secondary		1.08^*^ [1.02, 1.15]		1.44^***^ [1.34, 1.54]
Higher		1.39^***^ [1.20, 1.61]		1.71^***^ [1.48, 1.99]
Marital status				
Never in union		1.00		1.00
Married		1.46^***^ [1.36, 1.58]		1.39^***^ [1.28, 1.51]
Cohabiting		0.95 [0.88, 1.03]		0.91^*^ [0.83, 0.99]
Widowed		1.12 [0.96, 1.29]		1.46^***^ [1.25, 1.70]
Divorced		0.70^***^ [0.62, 0.79]		0.96 [0.83, 1.10]
Separated		0.95 [0.85, 1.06]		1.36^***^ [1.21, 1.53]
Place of delivery				
Home		1.00		1.00
Health facility		1.49^***^ [1.37, 1.61]		1.60^***^ [1.48, 1.74]
Other		1.69^***^ [1.49, 1.92]		1.71^***^ [1.50, 1.94]
Assistance during delivery				
No		1.00		1.00
Yes		2.66^***^ [2.45, 2.90]		1.88^***^ [1.72, 2.04]
Wealth index				
Poorest			1.00	1.00
Poorer			1.40^***^ [1.32, 1.48]	1.26^***^ [1.20, 1.34]
Middle			2.05^***^ [1.91, 2.19]	1.66^***^ [1.55, 1.78]
Richer			3.28^***^ [3.03, 3.55]	2.35^***^ [2.17, 2.55]
Richest			6.74^***^ [6.09, 7.46]	3.91^***^ [3.54, 4.33]
Sex of household head				
Male			1.00	1.00
Female			0.85^***^ [0.81,0.89]	0.83^***^ [0.79, 0.88]
Place of residence				
Urban			1.00	1.00
Rural			1.80^***^ [1.65, 1.97]	1.92^***^ [1.76, 2.10]
Subregion				
Central			1.00	1.00
Eastern			0.27^***^ [0.25, 0.30]	0.32^***^ [0.29, 0.34]
Southern			0.46^***^ [0.42, 0.51]	0.32^***^ [0.29, 0.35]
Western			1.84^***^ [1.69, 2.01]	1.71^***^ [1.57, 1.87]
Random effect model				
PSU variance (95% CI)	0.289 [0.243, 0.344]	0.215 [0.181, 0.256]	0.359 [0.298, 0.432]	0.331 [0.272, 0.401]
ICC	0.081	0.061	0.098	0.091
Wald chi-square	Reference	3361.90 (<0.001)	3348.77 (<0.001)	5156.21 (<0.001)
Model fitness				
Log-likelihood	−109357.19	−100873.8	−97306.214	−93012.537
AIC	218718.4	201795.6	194634.4	186091.1
Total weighted sample	162,500	162,500	162,500	162,500
Number of clusters	850	850	850	850

aOR, adjusted odds ratios; CI = confidence interval; ^*^p < 0.05, ^**^p < 0.01, ^***^p < 0.001; 1 = Reference category; PSU, primary sampling unit; ICC, intra-class correlation; AIC, Akaike’s information criterion.

Compared to adolescent girls, older women had a higher probability of registering their children’s birth. Children born to mothers with primary [aOR = 1.17; 95%CI = 1.11, 1.24], secondary [aOR = 1.44; 95%CI = 1.34, 1.54], or higher education [aOR = 1.71; 95%CI = 1.48, 1.99] were more likely to have been registered than those born to mothers who had no formal education. The odds of birth registration was higher among children born to married [aOR = 1.39; 95%CI = 1.28, 1.51], widowed [aOR = 1.46; 95%CI = 1.25, 1.70], and separated women [aOR = 1.36; 95%CI = 1.21, 1.53] compared to women who had never been in a union.

Also, children born in health facilities were more likely to have been registered [aOR = 1.60; 95%CI = 1.48, 1.74] than those born at home. The odds of birth registration was significantly higher among those who received assistance during delivery [aOR = 1.88; 95%CI = 1.72, 2.04] and those in rural areas [aOR = 1.92; 95%CI = 1.76, 2.10]. Increasing wealth index was associated with increasing likelihood of birth registration with the highest odds among those in the richest wealth index [aOR = 3.91; 95%CI =3.54, 4.33]. However, the likelihood birth registration was lower in female-headed households [aOR = 0.83; 95%CI = 0.79, 0.88] relative to those in male-headed households. Children in the Western sub-region were more likely to be registered [aOR = 1.71; 95%CI = 1.57, 1.87] than those in the Central Africa.

##### Random effect results

The random effect results show the variations in birth registration attributed to the model compositions. Results in Model O showed that the primary sampling unit clusters accounted for approximately 29% of the birth registration in SSA (σ^2^ = 0.289, 95% CI = 0.243–0.344). In the same model, the variation between the clusters contributed to 8.1% of the total variation of birth registration (ICC = 0.081). We observed that the between-cluster variance decreased to 6.1% (ICC = 0.061) in Model I then increased again to approximately 10% (ICC = 0.098) in Model II and finally reduced to 9.1% in the last model. These variations in the ICC value show that the differences in the factors at the household/community level accounted for much of the variations in birth registration relative to the individual level. Additionally, Model III had the lowest AIC (186091.1). Hence, Model III was selected as the best-fitted model.

## Discussion

We examined the predictors of birth registration among children in SSA. We observed a birth registration coverage of 48.32%, which is lower when compared to other jurisdictions such as India that has a coverage of 77.2% (18). Moreover, the observed coverage is lower than what has been reported in some individual SSA countries including Madagascar (79.9%), Sierra Leone (78%), and Ghana (62.5%) (19). Our study also shows the existence of significant sub-regional differences in the odds of childbirth registrations. Except for those in the Western region of SSA, the remaining sub-regions had lower odds of registering childbirths when compared to those in the Central region of SSA. It is unclear the reasons for the sub-regional variations. However, they may reflect a need for specific interventions and policies for the unique birth registration regimes in the respective sub-regions.

Our study found that older children had the highest odds of being registered compared to younger children. This result is corroborated by a study conducted in Zimbabwe that found a higher likelihood of birth registration among older children (3). We postulate that the lower odds of birth registration among younger children could be explained by the perspective that at the age below 1 year, the child would not have started schooling. Hence, parents may not see the importance of birth registration at that moment. However, these births are likely to be registered when children are about to enter school (3).

Having some level of formal education was associated with higher odds of birth registration. Similar findings have been reported in Ghana (9), India (18), and Nigeria (20). Education is expected to bring benefits, including the improvement in both the quantity and quality of available information over time. Hence, mothers who have completed formal education are better equipped to access institutional healthcare, have greater exposure to media, and possess greater knowledge of the birth registration process (18). These characteristics make them more likely to register their children’s births.

Contrary to Kumar et al.’s study (18) which found no significant association between marital status and birth registrations, our study showed that being in a marital union was significantly associated with higher odds of birth registration. Probably, married women are more likely to be supported by their partners to register the child’s birth—while those who have never been in a marital union may have to bear the total cost of being involved in accessing birth registration centers. Moreover, in most sub-Saharan African settings, the cultural norm is for the child to take the family name of the father (3). Thus, explaining the high odds of birth registration among those who were married and those who had ever been married (widowed and separated).

Giving birth in a health facility was associated with a higher probability of birth registration compared to home birth deliveries. Relatedly, those who received some assistance during the birth delivery were more likely than those who did not receive such assistance to register their children’s births. The results align with earlier reports from Zimbabwe (3) and SSA (6). Primarily, this association could be explained from the point that having an institutional birth delivery creates an opportunity for mothers to receive information about the benefits of birth registration, and possibly link them to birth registration centers (3).

The study also shows a positive association between wealth index and birth registration. That is, the higher the wealth index, the greater the odds of birth registration. Analogous findings have been reported in SSA (6) and India (18). Accessing birth registration centers is usually characterized by some economic challenges relating to the cost of transportation and other ancillary costs. Therefore, mothers in affluent households tend to have adequate financial resources to offset any associated cost of birth registration.

Relatedly, our result indicates that rural residency was associated with a higher chance of registering births—a finding that contradicts with a study conducted in SSA (6). A plausible explanation for this may have to do with issues of accessibility. Birth registration centers in rural areas are often located at a considerable distance from residential areas (21), which poses difficulties for parents to access them due to limited transportation options. However, it is possible that the parents of children in the rural areas were empowered to overcome barriers in accessing health and social services such as birth registration. In addition, the children in the rural areas might have benefited from free birth registration exercise organized in their localities.

Finally, the study revealed that children born into female-headed households were less likely to be registered. Our result is in contrast to an earlier study, involving 93 countries in low-and middle-income countries that found the sex of household heads as not being consistently associated with the birth registration of children (22). We posit that the observed association could be due to several reasons. One such plausibility is the point that female-headed households may lack the necessary economic resources which tends to create difficulties in terms of accessibility and affordability of registering their child’s birth. Another explanation could be that fathers may be considered as the primarily responsible individuals for birth registration, leading to additional bureaucratic challenges or confusion for households headed by females in the absence of a male parent.

## Policy implications

This study bears some policy implications. First, the significant differences in birth registration by sub-region imply that a straight jacket approach to resolving the lapses in childbirth registrations in SSA would prove futile. Rather, specific policies must be implemented to meet the needs and contextual environment of the respective sub-regions. The results also underscore a need for women empowerment to help alleviate any challenges they might face in the quest to register their children. Also, encouraging more birth deliveries at a health facility would be relevant in raising parents’ awareness of the need and benefits of registering their child’s birth.

## Strengths and limitations

Appropriate statistical analyses were conducted to arrive at the results. Nevertheless, there are some inherent limitations pertaining to the methodology. The study design used does not offer the opportunity to establish any sort of causality between the explanatory variables and the likelihood of registering a child’s birth. Due to the reliance on secondary data, important variables such as cultural norms and expectations could not be assessed in relation to their role as a predictor of childbirth registration.

## Conclusion

There is low childbirth registration coverage in SSA. The predictors of this phenomenon were the child’s age, maternal level of education, wealth index, place of residence, sub-region, maternal age, place of delivery, assistance during elivery, marital status, and sex of household head. Interventions and policies developed to improve childbirth registration coverage in SSA should prioritize mothers with no formal education, those who deliver at home, those with low socioeconomic status, those living in female headed household, and adolescent mothers.

## Data availability statement

Publicly available datasets were analyzed in this study. This data can be found at: http://dhsprogram.com/data/available-datasets.cfm.

## Ethics statement

Ethics approval was not required for this study since the data is secondary and is available in the public domain. More details regarding DHS data and ethical standards are available at: http://goo.gl/ny8T6X.

## Author contributions

SY conceived the study, supervised the study, and attest that all listed authors meet authorship criteria and that no others meeting the criteria have been omitted. RA, JO, A-AS, BA, EB, and SY contributed to interpretation of data, revised the article critically for important intellectual content, and approved the final version of the manuscript. All authors contributed to the article and approved the submitted version.

## Conflict of interest

SY is an editorial board member of this journal. A-AS and BOA were employed by REMS Consult Limited.

The remaining authors declare that the research was conducted in the absence of any commercial or financial relationships that could be construed as a potential conflict of interest.

## Publisher’s note

All claims expressed in this article are solely those of the authors and do not necessarily represent those of their affiliated organizations, or those of the publisher, the editors and the reviewers. Any product that may be evaluated in this article, or claim that may be made by its manufacturer, is not guaranteed or endorsed by the publisher.
